# Supplementary material for: Mechanisms governing the pioneering and redistribution capabilities of the non-classical pioneer PU.1
Source: Nat Commun. 2020 Jan 21;11:402. doi: 10.1038/s41467-019-13960-2 (PMC6972792; doi:10.1038/s41467-019-13960-2)
Supplement: Supplementary file 7 — Source data [file 41467_2019_13960_MOESM7_ESM.zip › Source_Data/FigureS3/FigureS3J_MotifScanOutput/homerResults/motif2.similar.html]

motif2

## Information for motif2

C
G
A
T
C
T
A
G
G
C
A
T
C
A
G
T
C
A
G
T
C
T
G
A
G
A
T
C
G
C
T
A
A
G
C
T
C
G
T
A
C
T
A
G
  
Reverse Opposite:  

G
A
T
C
G
C
A
T
T
C
G
A
C
G
A
T
C
T
A
G
A
G
C
T
G
T
C
A
G
T
C
A
C
G
T
A
A
G
T
C
G
C
T
A
  

|  |  |
| --- | --- |
| p-value: | 1e-636 |
| log p-value: | -1.465e+03 |
| Information Content per bp: | 1.609 |
| Number of Target Sequences with motif | 5974.0 |
| Percentage of Target Sequences with motif | 45.27% |
| Number of Background Sequences with motif | 7376.7 |
| Percentage of Background Sequences with motif | 20.21% |
| Average Position of motif in Targets | 366.1 +/- 307.7bp |
| Average Position of motif in Background | 318.0 +/- 199.7bp |
| Strand Bias (log2 ratio + to - strand density) | 0.0 |
| Multiplicity (# of sites on avg that occur together) | 1.40 |
| Motif File: | file (matrix) reverse opposite |

### Similar de novo motifs found

|  |  |  |  |  |  |  |  |
| --- | --- | --- | --- | --- | --- | --- | --- |
| Rank | Match Score | Redundant Motif | P-value | log P-value | % of Targets | % of Background | Motif file |
| 1 | 0.978 | T C A G G A T C C G A T T C A G C G T A C T A G A G C T G T C A G T C A C G T A A G T C G T C A | 1e-635 | -1462.212013 | 42.39% | 18.00% | motif file (matrix) |
| 2 | 0.975 | C A G T T C A G A C G T C G A T C A G T C T G A G A T C G C A T A G T C C G T A | 1e-634 | -1460.444288 | 48.50% | 22.85% | motif file (matrix) |
| 3 | 0.946 | G T A C C G A T T C G A C G A T A C T G A G T C C G T A C G T A C G T A A G T C C G T A T C G A G C T A | 1e-564 | -1299.781997 | 38.20% | 16.00% | motif file (matrix) |
| 4 | 0.915 | T A C G C G T A A C T G A G C T G T C A C G T A C G T A A G T C C G T A | 1e-557 | -1283.180297 | 51.41% | 26.77% | motif file (matrix) |
| 5 | 0.974 | C G A T C T A G A C G T A C G T A C G T C T G A G A T C G C T A A G T C C G T A C T A G C A G T C T A G G T C A | 1e-530 | -1221.013319 | 37.02% | 15.69% | motif file (matrix) |
| 6 | 0.935 | C G A T C A T G G A C T G T C A G T C A T G C A A G T C G C T A | 1e-502 | -1157.568529 | 50.13% | 26.78% | motif file (matrix) |
| 7 | 0.884 | A C T G A G T C C G T A C G T A C G T A A G T C C G T A | 1e-497 | -1144.671698 | 50.96% | 27.63% | motif file (matrix) |
| 8 | 0.854 | C G A T C T A G A G C T A C G T A C G T C T G A G C T A G A T C A G T C | 1e-251 | -580.004659 | 35.85% | 20.62% | motif file (matrix) |
| 9 | 0.832 | A T G C G C A T A C T G C G A T T C A G G A T C C G T A T G C A T C G A A T G C G T C A | 1e-245 | -566.088910 | 42.30% | 26.34% | motif file (matrix) |
| 10 | 0.793 | C G T A G A T C A C G T C T A G A C G T A C G T C T A G C G T A A G T C G C T A A C G T C G T A | 1e-147 | -339.452709 | 11.20% | 4.47% | motif file (matrix) |
| 11 | 0.825 | C G A T T C A G G A T C G C A T C T G A T C G A C G T A A G T C C G T A C T G A C G T A A G T C C G T A | 1e-147 | -338.774443 | 21.55% | 11.97% | motif file (matrix) |
| 12 | 0.802 | G C A T G T A C C G A T C T A G A G C T C A G T T A C G G C T A G T A C G C A T A G T C G C T A C T G A A C T G | 1e-116 | -268.908639 | 9.13% | 3.68% | motif file (matrix) |
| 13 | 0.753 | C G A T A C T G A C G T A G T C C T G A C G T A A G T C C T G A | 1e-110 | -254.602620 | 13.68% | 6.98% | motif file (matrix) |
| 14 | 0.761 | T A C G G C A T C A G T A C T G G T C A A T G C C G T A T G C A C G T A A T G C C G T A T C A G | 1e-105 | -243.257733 | 18.66% | 10.95% | motif file (matrix) |
| 15 | 0.780 | T G A C C T A G G A C T G A T C C G T A C T G A C G T A A G T C G T C A | 1e-101 | -233.730164 | 44.72% | 34.13% | motif file (matrix) |
| 16 | 0.750 | G C A T A G T C C G T A C G T A C G T A A G T C C G T A | 1e-85 | -195.925622 | 32.95% | 24.04% | motif file (matrix) |
| 17 | 0.798 | A C G T C T A G C G A T A G C T A G C T C T A G C A G T | 1e-84 | -194.148440 | 53.29% | 43.40% | motif file (matrix) |
